# Diagnosis of Acute Heart Failure in the Emergency Department: An Evidence-Based Review

**DOI:** 10.5811/westjem.2019.9.43732

**Published:** 2019-10-24

**Authors:** Brit Long, Alex Koyfman, Michael Gottlieb

**Affiliations:** * Brooke Army Medical Center, Department of Emergency Medicine, Fort Sam Houston, Texas; † University of Texas Southwestern Medical Center, Department of Emergency Medicine, Dallas, Texas; ‡ Rush University Medical Center, Department of Emergency Medicine, Chicago, Illinois

## Abstract

Heart failure is a common presentation to the emergency department (ED), which can be confused with other clinical conditions. This review provides an evidence-based summary of the current ED evaluation of heart failure. Acute heart failure is the gradual or rapid decompensation of heart failure, resulting from either fluid overload or maldistribution. Typical symptoms can include dyspnea, orthopnea, or systemic edema. The physical examination may reveal pulmonary rales, an S3 heart sound, or extremity edema. However, physical examination findings are often not sensitive or specific. ED assessments may include electrocardiogram, complete blood count, basic metabolic profile, liver function tests, troponin, brain natriuretic peptide, and a chest radiograph. While often used, natriuretic peptides do not significantly change ED treatment, mortality, or readmission rates, although they may decrease hospital length of stay and total cost. Chest radiograph findings are not definitive, and several other conditions may mimic radiograph findings. A more reliable modality is point-of-care ultrasound, which can facilitate the diagnosis by assessing for B-lines, cardiac function, and inferior vena cava size. These modalities, combined with clinical assessment and gestalt, are recommended.

## INTRODUCTION

Acute heart failure (AHF) is a gradual or rapid decompensation in heart failure (HF) requiring urgent management.^1^–^4^ The condition covers a large spectrum of disease, ranging from mild exacerbations with gradual increases in edema to cardiogenic shock. HF affects close to six million people in the United States (U.S.) and increases in prevalence with age.^6^–^11^ Currently, the emergency department (ED) initiates the evaluation and treatment of over 80% of patients with AHF in the U.S.^12^–^17^ As the population ages, increasing numbers of patients with HF will present to the ED for evaluation and management. However, making the correct diagnosis can be challenging due to the broad differential diagnosis associated with presenting symptoms and variations in patient presentations.

Over one million patients are admitted for HF in the U.S. and Europe annually.^6^–^11^,^16^–^20^ In the U.S. population, people have a 20% risk of developing HF by 40 years of age.^21^–^25^ HF is more common in males until the age of 65, at which time males and females are equally affected.^25^–^28^ Patients with HF average at least two hospital admissions per year.^25^,^29^,^30^ Among patients who are admitted with AHF, over 80% have a prior history of HF, referred to as decompensated heart failure.^20^–^23^ De novo HF is marked by no previous history of HF combined with symptom appearance after an acute event.^3^,^4^,^19^,^23^ Mortality in patients with HF can be severe, with up to half of all patients dying within five years of disease diagnosis.^20^,^21^,^25^ Other studies have found that post-hospitalization mortality rates at 30 days, one year, and five years are 10.4%, 22%, and 42.3%, respectively.^23^–^27^ AHF expenditures approach $39 billion per year, which is expected to almost double by 2030.^31^,^32^

## METHODS

We searched PubMed and Google Scholar for articles using the keywords “heart failure” and “emergency.” We included retrospective studies, prospective studies, systematic reviews and meta-analyses, clinical guidelines, and narrative reviews focusing on diagnosis of HF including history and physical examination, biomarkers, electrocardiogram (ECG), and imaging. The literature search was restricted to studies published in English. Emergency physicians with experience in critical appraisal of the literature reviewed all of the articles and decided which studies to include for the review by consensus, with a focus on emergency medicine-relevant articles. A total of 124 articles were selected for inclusion in this review.

## DISCUSSION

### Anatomy and Pathophysiology

Normal cardiac physiology is dependent on appropriately functioning ventricular contraction, ventricular wall structural integrity, and valvular competence.^28^,^33^,^34^ At normal functional status, a person’s stroke volume (SV) is approximately one milliliter (mL) per kilogram for every heartbeat.^28^,^33^–^36^ SV is dependent upon the preload (defined as the amount of myocardial muscle fiber stretch at the end of ventricular filling), afterload (defined as the amount of vascular resistance the ventricle must overcome), and contractility (defined as the strength of the myocardial contraction). In patients with HF, left ventricular (LV) dysfunction can be due to impaired LV contraction and ejection (systolic dysfunction), impaired relaxation and filling (diastolic dysfunction), or a combination of both.^28^,^33^

An alternate way of defining this would be by the effect on ejection fraction (EF). HF with preserved EF refers to patients with an EF > 50%, while HF with reduced EF refers to patients with an EF < 40%. Borderline preserved EF is defined by HF with an EF of 41–50%.^3^,^4^,^17^,^18^,^29^ The most common form is HF with reduced EF, which is primarily related to a decrease in the functional myocardium (typically associated with ischemic disease or a prior myocardial infarction).^3^,^4^,^34^ Additional causes include excessive pressure overload from hypertension, valvular incompetence, and cardiotoxic medications. HF with preserved EF occurs due to impaired ventricle relaxation and filling, which accounts for 30–45% of all HF cases.^22^,^23^,^33^,^37^,^38^ This form of HF results in increased end-systolic and diastolic volumes and pressures and is most commonly associated with chronic hypertension, coronary artery disease, diabetes mellitus, cardiomyopathy, and valvular disease. Both systolic and diastolic HF can present with similar symptoms due to elevated, left-sided intracardiac pressures and pulmonary congestion.^25^,^28^,^33^–^36^

Right ventricular failure most commonly results from LV failure. As the right side of the heart fails, increased pressure in the vena caval system elevates pressure in the venous system of the gastrointestinal tract, liver, and extremities, resulting in edema, jugular venous distension, hepatomegaly, bloating, abdominal pain, and nausea.^25^,^28^,^33^,^34^ High-output HF is associated with normal or greater-than-normal cardiac output and decreased systemic vascular resistance.^34^–^38^ The associated decrease in afterload reduces arterial blood pressure and also activates neurohormones, which increase salt and water retention. Diseases that may result in high-output HF include anemia, large arteriovenous fistula or multiple small fistulas, severe hepatic or renal disease, hyperthyroidism, beriberi disease, and septic shock.^36^–^38^

In AHF, peripheral vascular flow and end-organ perfusion decrease, causing the body to compensate by neurohormonal activation (ie, the renin-angiotensin system), ventricular remodeling, and release of natriuretic peptides.^25^,^28^,^34^,^35^ These mechanisms are chronically activated in HF, but worsen during acute exacerbations, resulting in hemodynamic abnormalities leading to further deterioration. Continued progression can result in a critical reduction to end-organ blood flow, leading to severe morbidity and mortality.^3^,^4^,^25^,^28^,^33^–^35^

### Heart Failure Classification

Patients with HF are classified into one of four classes, primarily determined by daily function, using the New York Heart Association, American College of Cardiology/American Heart Association, or European Society of Cardiology Guidelines (Table 1).^17^,^18^,^39^–^41^ These systems help determine the appropriate interventions to reduce the likelihood of developing severe LV dysfunction, thereby reducing the patient’s potential morbidity and mortality.^3^,^4^,^17^,^18^,^34^ Other means of classification depend on the presence of cardiomyopathy or acute coronary syndrome (ACS). The Nohria-Stevenson classification for decompensated HF in the setting of cardiomyopathy uses perfusion and congestion, while the Killip and Forrester classification systems evaluate AHF in the setting of ACS.^12^,^17^,^18^,^39^–^45^ In general, short-term mortality is low for well-perfused groups and is higher in poorly-perfused patients.^12^,^17^,^18^,^39^–^45^

Unfortunately, these classification systems are not as useful for acute exacerbation of HF, thereby limiting their applicability in the ED setting. In the ED, classification is based upon the patient’s hemodynamic status, perfusion, and blood pressure.^3^,^4^,^30^,^42^ This differentiation can guide therapy and provides important prognostic information. Most patients are hypertensive or normotensive upon presentation.^16^–^22^ The hypertensive form (associated with a systolic blood pressure > 140 millimeters of mercury (mmHg) is commonly associated with pulmonary edema, which may occur rapidly (ie, flash pulmonary edema).^46^,^47^ In the normotensive progressive form, systemic edema is predominant.^16^–^22^,^30^ Hypotensive AHF is associated with end-organ hypoperfusion, while systemic and pulmonary edema is minimal. ACS can occur simultaneously with or exacerbate HF and requires emergent coronary angiography.^48^,^49^ Right-sided HF is associated with right ventricular dysfunction, leading to systemic venous congestion without pulmonary edema if the LV is not involved.^3^,^4^,^30^

### History and Physical Examination

Due to the complex pathophysiology involved in HF and multiple phenotypes (eg, low- vs high-output, preserved vs reduced EF, left-sided vs right-sided), the history and physical examination may vary. Patients with HF are heterogeneous in terms of the cardiac structure and function, the etiology of their HF, the precipitant of the AHF exacerbation, comorbidities, and current medications. Early diagnosis is vital, as a delay or misdiagnosis has been associated with an increased risk of adverse outcomes and death.^50^–^52^ Misdiagnosis occurs in up to one-third of patients upon initial presentation.^53^–^56^ While no single historical factor or examination finding can significantly reduce the likelihood of HF in isolation, initial clinical gestalt has been shown to have a sensitivity of 61% and specificity of 86% for the diagnosis.^57^,^58^

Risk factors for HF include hypertension, renal disease, heart disease, diabetes, male gender, older age, and obesity.^58^–^61^ In particular, advanced age, renal disease, and lower blood pressure are associated with increased mortality in AHF.^60^,^61^ Precipitating factors for AHF exacerbation can include cardiac and non-cardiac causes.^63^,^64^ Cardiac causes include uncontrolled hypertension, dietary or medication noncompliance, aortic dissection, dysrhythmias, and cardiac ischemia.^30^,^59^,^63^,^64^ Non-cardiac causes include pulmonary disease, endocrine disease, infection, worsening renal function, anemia, and medication side effects.^3^,^4^,^30^,^59^ Patients who are noncompliant with their diet and medications have been found to have a lower EF, higher brain-type natriuretic peptide (BNP) levels, and greater congestion when compared with their counterparts.^30^,^63^,^64^ Dysrhythmias are another frequent precipitating cause. Among those, atrial fibrillation is the most common.^17^,^18^,^21^,^29^ ACS is more commonly associated with de novo HF.^17^,^18^,^29^ Components of the history such as weight gain, dyspnea, chest pain, peripheral edema, substance abuse, new medications, past complications, prior hospitalizations, diet changes (eg, salt or fluid intake), and medication compliance are vital to determine the underlying etiology, and an identifiable trigger can be found in approximately 60% of patients.^58^–^62^

Acutely, the most common symptoms associated with AHF include paroxysmal nocturnal dyspnea (PND), orthopnea, and edema.^16^,^29^,^30^,^57^–^59^ The most common manifestation is dyspnea or edema from elevated LV filling pressures.^4^,^57^–^59^ However, the classic symptoms such as PND, dyspnea, and orthopnea demonstrate poor sensitivity and specificity (Table 2).^59^,^65^–^67^

On examination, an S3 heart sound has the highest specificity, ranging from 97.7–99%, but it has only 12.7% sensitivity.^53^,^54^,^57^–^59^ Additionally, an S3 heart sound can be difficult to detect in the ED setting, and inter-rater reliability can be poor.^3^,^4^,^59^ Hepato-jugular reflux and jugular venous distension possess a specificity of 93.4% and 87% and sensitivity 14.1% and 37.2%, respectively, for HF.^57^–^59^ Lung auscultation is also less reliable, as the presence of rales has a sensitivity of approximately 60% and a specificity approaching 70%.^57^–^59^ Lower extremity edema has a sensitivity of 50% and specificity 78%.^57^–^59^ A meta-analysis evaluating various signs and symptoms in patients with dyspnea found that no single sign or symptom was sufficiently able to rule out AHF, chronic obstructive pulmonary disease, asthma, or pulmonary embolism.^65^ However, elevated jugular venous pressure, third heart sound, and lung crepitations were strongly suggestive of a diagnosis of AHF.^65^

### Laboratory Testing

Laboratory assessment in the patient with suspected AHF can provide important diagnostic and prognostic information.^3^,^4^,^30^,^58^,^59^ Testing should include a complete blood count, basic metabolic panel with renal function testing, liver function testing, troponin, and a BNP level.^30^,^48^–^50^–^47^,^55^,^56^ Abnormalities in liver function are found in approximately 75% of patients with AHF and are associated with more severe disease.^30^,^69^ If the right ventricle is involved, bilirubin and alkaline phosphatase levels may be elevated, while left-sided disease is more commonly associated with elevated transaminase levels.^30^,^69^ Renal function is an important assessment, as it is a predictor of disease severity and mortality.^15^–^18^,^70^ Decreased glomerular filtration rate (GFR) is associated with increased length of in-hospital stay, short-term mortality, and long-term mortality.^17^,^18^,^70^–^72^ In patients with AHF, every 10 mL/minute decrease in GFR is associated with an increase in mortality of 7%.^71^,^72^

Troponin testing can assist in prognostication and in the detection of underlying ischemia as a potential inciting event for AHF. Elevated troponin levels are associated with higher re-hospitalization rates and 90-day mortality.^17^,^18^,^48^,^49^ Troponin elevation is common in AHF, as one study found elevated troponin levels in 98% of patients with diagnosed AHF, with 81% of the levels above the 99^th^ percentile.^73^ Other studies have suggested that this may be closer to 30–50%.^3^,^4^,^30^ However, an elevated troponin is not specific for ACS and may be seen with a variety of other causes, including demand ischemia and renal dysfunction.^17^,^18^,^48^–^50^

Natriuretic peptides (ie, BNP and NT-proBNP) may be a valuable adjunct when the provider is unclear of the diagnosis.^57^–^59^,^74^–^77^ BNP is produced by cardiac myocytes when exposed to significant myocardial stretch. Use of BNP and NT-proBNP may be sensitive, but not specific for the diagnosis of AHF. Levels less than 100 picograms (pg) per milliliter (mL) for BNP have demonstrated a sensitivity and specificity of 93.5% and 52.9%, respectively, with negative likelihood ratio (LR-) of 0.2.^57^–^59^ Using a 300 pg/mL cut-off for NT-proBNP demonstrates a LR− of 0.09.^59^ However, elevated levels only moderately increase the likelihood of AHF, as specificity improves to 72.9% with a value of 1550 pg/mL for NT-proBNP.^59^,^74^–^79^ A BNP level > 400 pg/mL or a NT-proBNP level > 900 pg/mL is consistent with AHF; however, in patients over the age of 75 years, the NT-proBNP level should be increased to 1800 pg/mL.^3^,^4^,^30^,^74^–^77^ Obesity can falsely lower the natriuretic peptides levels,^3^,^4^,^30^,^74^–^76^,^79^ while renal disease may falsely elevate levels (especially with GFR < 60 mL/min).^74^,^75^,^80^,^81^

Other conditions associated with elevations in natriuretic peptide levels include pulmonary embolism, pulmonary hypertension, valvular heart disease, and acute respiratory distress syndrome. BNP levels of 100–400 pg/mL and NT-proBNP levels of 300–900 pg/mL are non-specific and may require further testing.^74^–^77^,^82^–^87^ Although these biomarkers may assist in differentiation of other conditions, studies have not demonstrated improved patient-centered outcomes with use of natriuretic peptides.^86^–^88^ Observational trial data suggest natriuretic peptides demonstrate sensitivity over 90%, but specificity is poor.^80^,^88^–^92^ Data from randomized, controlled trials found that knowledge of the BNP levels did not significantly change the ED treatment, mortality, or readmission rates; however, it may decrease hospital length of stay and total cost.^76^,^93^–^99^

### Electrocardiogram

An ECG should be rapidly obtained to evaluate for the etiology or precipitating factors (eg, ACS, atrial fibrillation with rapid ventricular response, ventricular dysrhythmia).^3^,^4^,^26^,^57^,^59^ An ECG is unlikely to diagnose or exclude AHF in isolation.^57^,^59^,^100^,^101^ Prolonged QRS and junctional rhythms are associated with worse patient outcomes.^100^,^101^ Table 3 demonstrates ECG findings in AHF.^57^,^100^,^101^

### Imaging

Imaging is an important component in the patient with suspected heart failure. The most common modality used is the chest radiograph (CXR). Several findings suggest the diagnosis of heart failure on CXR, including cardiomegaly, central vascular congestion, and interstitial edema (Table 4).^17^,^18^,^41^,^102^ However, a normal CXR should not be used to exclude the diagnosis of AHF, as up to 20% of CXRs may appear normal in AHF.^4^,^102^–^106^ Studies evaluating physician accuracy with identifying AHF on CXR have demonstrated sensitivities of 59–74.5% and specificities of 86.3–96%.^59^,^103^–^105^ While CXR should not be used to exclude AHF, it can be valuable for identifying alternate disease processes that may mimic AHF.^3^,^4^,^102^–^105^

Bedside ultrasound can be valuable for diagnosing AHF, with high specificity and positive likelihood ratios (Table 5). Ultrasound can be used to evaluate for B-lines, pleural effusions, inferior vena cava size and respiro-phasic variability, and cardiac contractility.^59^,^106^–^108^ B-lines are vertical artifacts that result from sound wave reverberation through fluid-filled pulmonary interstitium. The presence of greater than three B-lines in two bilateral lung zones defines a positive lung ultrasound examination.^56^,^106^–^113^ The number of lung zones examined varies in the literature, with eight thoracic lung zones used in the initial lung ultrasound protocols, while newer studies have used four or six lung zones. B-lines demonstrate high sensitivity and specificity for interstitial edema,^59^,^107^,^108^ while the identification of pleural effusions is not as helpful.^59^

Assessment of EF on ultrasound may be assessed with visual assessment or quantitative measurements. Qualitative visual estimation is made by assessing the inward movement of the interventricular septum and inferior wall of the LV during systole.^59^,^106^–^113^ E-point septal separation (EPSS) is a quantitative measurement assessing the distance between the anterior mitral valve leaflet and ventricular septum. An EPSS measurement > 7 mm is suggestive of an EF < 50%.^111^–^114^ Ultrasound can also estimate intravascular volume through the measurement of inferior vena cava diameter and percentage change during the respiratory cycle. However, diagnostic performance is controversial, with many confounding factors and a wide range of sensitivities and specificities.^115^–^117^ One study found that by using a combination of lung, cardiac, and inferior vena cava ultrasound, the authors were able to improve diagnostic accuracy by 20%.^118^ Others have suggested that combining CXR with ultrasound may increase the sensitivity and specificity for diagnosing AHF.^103^

### Disposition

Due to the heterogenous nature of heart failure, disposition may be challenging. The majority of patients presenting to the ED in the U.S. with AHF are admitted.^12^–^14^ Patients with hemodynamic instability or critical illness should be admitted to an intensive care unit, and patients with newly diagnosed HF may benefit from admission for further evaluation and management.^17^,^18^,^21^,^119^ Other patients who may require admission include those with poor response to medical treatment or inability to obtain follow-up, significant electrolyte abnormalities, elevated blood urea nitrogen or creatinine, or ischemia on ECG or biomarker testing.^120^ In those with prior history of HF and the absence of the aforementioned items, risk stratification tools such as the Emergency Heart Failure Mortality Risk Grade or the Ottawa Heart Failure Risk Score may be able to identify a select subset of low-risk patients, but these scoring systems require further validation.^120^–^124^

## CONCLUSION

Heart failure is a common presentation to the ED, which can be confused with other clinical conditions. Acute heart failure refers to the gradual or rapid decompensation of heart failure, resulting from either fluid overload or maldistribution. Typical symptoms can include dyspnea, orthopnea, or edema. The physical examination may reveal pulmonary rales, an S3 heart sound, or extremity edema. Laboratory studies should include an electrocardiogram, complete blood count, basic metabolic profile, coagulation studies, troponin, brain natriuretic peptide, and a chest radiograph. Point-of-care ultrasound can facilitate the diagnosis by assessing for B-lines, cardiac function, and inferior vena cava size. Understanding the diagnostic approach can improve the diagnostic accuracy and allow for more rapid initiation of the correct intervention.

## Figures and Tables

**Table 1:** Heart failure classification systems.^17^,^18^,^39^–^41^

NYHA	ACC/AHA	ESC guidelines
Class I: No symptoms with ordinary activity.	Stage A: Patient is at high risk for developing HF.	1. Heart failure with reduced ejection fraction (< 40%).
Class II: Slight limitation with physical activity. No issues at rest, but physical activity can result in fatigue, palpitations, dyspnea, or angina.	Stage B: Patient has structural heart disorder but no symptoms of HF.	2. Heart failure with mid-range ejection fraction (40–49%).
Class III: Severe limitation in physical activity. Comfortable at rest. However, less than normal physical activity results in fatigue, palpitations, dyspnea, or angina.	Stage C: Patient has past or current symptoms of HF with underlying structural heart disease.	3. Heart failure with preserved ejection fraction (> 50%).
Class IV: Unable to perform physical activity without discomfort. Symptoms may be present at rest.	Stage D: Patient has end-stage disease and requires specialized treatment strategies.	

*NYHA*, New York Heart Association; *ACC/AHA*, American College of Cardiology/American Heart Association; *ESC*, European Society of Cardiology; *HF*, heart failure.

**Table 2:** History and examination findings in acute heart failure.^59^

Finding	Sensitivity (95% CI)	Specificity (95% CI)	+LR (95% CI)	−LR (95% CI)
Orthopnea	52.1 (50.1–54.0)	70.5 (68.8–72.1)	1.9 (1.4–2.5)	0.74 (0.64–0.85)
PND	46.2 (43.7–48.6)	73.9 (71.9–75.9)	1.6 (1.2–2.1)	0.79 (0.71–0.88)
Dyspnea at rest	54.6 (51.2–58.0)	49.6 (46.9–52.3)	1.1 (0.9–1.4)	0.88 (0.74–1.04)
No productive cough	82.0 (79.6–84.4)	25.8 (23.5–28.2)	1.13 (1.02–1.26)	0.6 (0.5–0.8)
History of CHF	55.5 (53.9–57.1)	80.2 (79.0–81.3)	2.7 (2.0–3.7)	0.58 (0.49–0.68)
History of MI	31.8 (29.7–33.9)	87.1 (85.8–88.3)	2.1 (1.8–2.5)	0.82 (0.76–0.89)
History of AF	30.2 (27.4–33.2)	85.3 (82.8–87.5)	2.1 (1.6–2.9	0.82 (0.71–0.93)
History of CAD	46.6 (44.5–48.7)	76.2 (74.6–77.7)	2.0 (1.7–2.4)	0.71 (0.64–0.79)
History of DM	28.8 (27.4–30.4)	81.7 (80.4–82.8)	1.5 (1.3–1.7)	0.89 (0.84–0.94)
History of CRD	32.0 (29.4–34.6)	91.4 (90.0–92.7)	3.4 (2.7–4.5)	0.75 (0.71–0.80)
History of HTN	66.9 (65.5–68.3)	50.7 (49.4–52.1)	1.3 (1.3–1.4)	0.62 (0.53–0.73)
S3	12.7 (11.5–14.0)	97.7 (97.2–98.2)	4.0 (2.7–5.9)	0.91 (0.88–0.95)
JVD	37.2 (35.7–38.7)	87.0 (85.9–88.0)	2.8 (1.7–4.5)	0.76 (0.69–0.84)
Hepato-jugular reflex	14.1 (11.9–16.6)	93.4 (91.2–95.2)	2.2 (1.3–3.7)	0.91 (0.88–0.94)
Leg edema	51.9 (50.5–53.4)	75.2 (74.0–76.4)	1.9 (1.6–2.3)	0.68 (0.61–0.75)
Rales	62.3 (60.8–63.7)	68.1 (66.7–69.4)	1.8 (1.5–2.1)	0.60 (0.51–0.69)
Wheeze	22.3 (20.9–23.8)	64.0 (62.5–65.4)	0.6 (0.5–0.8)	1.19 (1.10–1.30)
No fever	92.4 (90.9–93.8)	20.6 (18.8–22.5)	1.14 (1.02–1.27)	0.4 (0.3–0.6)
Murmur	27.8 (25.8–29.9)	83.2 (81.6–84.8)	1.9 (0.9–3.9)	0.93 (0.79–1.08)

*CI*, confidence interval; *PND*, paroxysmal nocturnal dyspnea; *CHF*, congestive heart failure; *MI*, myocardial infarction; *AF*, atrial fibrillation; *CAD*, coronary artery disease; *DM*, diabetes mellitus; *CRD*, chronic respiratory disease; *HTN*, hypertension; *JVD*, jugular venous distension.

**Table 3:** Electrocardiogram findings in acute heart failure.^59^

Finding	Sensitivity (95% CI)	Specificity (95% CI)	+LR (95% CI)	−LR (95% CI)
Ischemic changes	34.0 (29.8–38.4)	84.2 (81.2–86.9)	2.9 (1.2–7.1)	0.78 (0.73–0.84)
T-wave inversion	10.0 (7.5–13.0)	95.9 (92.3–98.1)	2.4 (1.2–4.8)	0.94 (0.90–0.98)
ST depression	5.6 (3.9–7.7)	96.5 (94.2–98.1)	2.0 (1.0–3.8)	0.97 (0.95–1.00)
ST elevation	5.2 (2.1–10.5)	91.8 (83.8–96.6)	0.6 (0.2–1.7)	1.03 (0.96–1.11)
Atrial fibrillation	20.5 (18.3–22.9)	89.9 (87.9–91.7)	2.2 (1.4–3.5)	0.88 (0.85–0.91)
Normal sinus rhythm	55.4 (50.9–60.0)	17.8 (15.1–20.8)	0.7 (0.5–0.9)	2.88 (1.26–6.57)

*CI*, confidence interval; *LR*, likelihood ratio.

**Table 4:** Chest radiograph findings in acute heart failure.^59^

Finding	Sensitivity (95% CI)	Specificity (95% CI)	+LR (95% CI)	−LR (95% CI)
Kerley B lines	9.2 (6.5–12.5)	98.8 (97.3–99.6)	6.5 (2.6–16.2)	0.88 (0.69–1.13)
Interstitial edema	31.1 (28.2–34.2)	95.1 (93.6–96.3)	6.4 (3.4–12.2)	0.73 (0.68–0.78)
Cephalization	44.7 (41.1–48.4)	94.6 (92.6–96.3)	5.6 (2.9–10.4)	0.53 (0.39–0.72)
Alveolar edema	5.7 (4.7–6.9)	98.9 (98.4–99.3)	5.3 (3.3–8.5)	0.95 (0.94–0.97)
Pulmonary edema	56.9 (54.7–59.1)	89.2 (87.9–90.4)	4.8 (3.6–6.4)	0.48 (0.39–0.58)
Pleural effusion	16.3 (13.7–19.2)	92.8 (90.4–94.7)	2.4 (1.6–3.6)	0.89 (0.80–0.99)
Cardiomegaly	74.7 (72.9–76.5)	61.7 (59.4–63.9)	2.3 (1.6–3.4)	0.43 (0.36–0.51)

*CI*, confidence interval; *LR*, likelihood ratio.

**Table 5:** Bedside ultrasound findings in acute heart failure.^59^,^107^

Finding	Sensitivity (95% CI)	Specificity (95% CI)	+LR (95% CI)	−LR (95% CI)
Positive B lines	94.1 (81.3–98.3)	92.7 (90.9–94.3)	12.4 (5.7–26.8)	0.06 (0.02–0.22)
Pleural effusion	63.5 (50.4–75.3)	71.7 (61.4–80.6)	2.0 (1.4–2.8)	0.49 (0.22–1.10)
Reduced EF	80.6 (72.9–86.9)	80.6 (74.3–86.0)	4.1 (2.4–7.2)	0.24 (0.17–0.35)
Increased LV end-diastolic dimension	79.6 (65.7–89.7)	68.6 (50.7–83.1)	2.5 (1.5–4.2)	0.30 (0.16–0.54)
Restrictive mitral pattern	81.5 (68.6–90.7)	90.1 (80.7–95.9)	8.3 (4.0–16.9)	0.21 (0.12–0.36)

*CI*, confidence interval; *LR*, likelihood ratio; *EF*, ejection fraction; *LV*, left ventricular.

## References

[1] American Heart Association Heart failure.

[2] NHLBI What is heart failure?.

[3] Kuo DC, Peacock WF (2015). Diagnosing and managing acute heart failure in the emergency department. Clin Exp Emerg Med.

[4] Hunter BR, Martindale J, Abdel-Hafez O (2017). Approach to acute heart failure in the emergency department. Prog Cardiovasc Dis.

[5] Christ M, Stork S, Dorr M, Trend HF Germany Project (2016). Heart failure epidemiology 2000–2013: insights from the German Federal Health Monitoring System. Eur J Heart Fail.

[6] Gabet A, Juillière Y, Lamarche-Vadel A (2015). National trends in rate of patients hospitalized for heart failure and heart failure mortality in France, 2000–2012. Eur J Heart Fail.

[7] Omersa D, Farkas J, Erzen I, Lainscak M (2016). National trends in heart failure hospitalization rates in Slovenia 2004–2012. Eur J Heart Fail.

[8] Schmidt M, Ulrichsen SP, Pedersen L (2016). Thirty-year trends in heart failure hospitalization and mortality rates and the prognostic impact of co-morbidity: a Danish nationwide cohort study. Eur J Heart Fail.

[9] Ambrosy AP, Fonarow GC, Butler J (2014). The global health and economic burden of hospitalizations for heart failure: lessons learned from hospitalized heart failure registries. J Am Coll Cardiol.

[10] Mozaffarian D, Benjamin EJ, Go AS, Arnett DK, American Heart Association Statistics Committee; Stroke Statistics Subcommittee (2016). Heart disease and stroke statistics—2016 update. A report from the American Heart Association. Circulation.

[11] Crespo-Leiro MG, Anker SD, Maggioni AP (2016). Heart Failure Association (HFA) of the European Society of Cardiology (ESC). European Society of Cardiology Heart Failure Long-Term Registry (ESC-HF-LT): 1-year follow-up outcomes and differences across regions. Eur J Heart Fail.

[12] Pang PS, Collins SP, Miro O (2015). The role of the emergency department in the management of acute heart failure: an international perspective on education and research. Eur Heart J Acute Cardiovasc Care.

[13] Storrow AB, Jenkins CA, Self WH (2014). The burden of acute heart failure on U.S. emergency departments. JACC Heart Fail.

[14] Pang PS, Collins SP (2017). Acute heart failure in the emergency department: just a one night stand?. Acad Emerg Med.

[15] Bleumink GS, Knetsch AM, Sturkenboom MC (2004). Quantifying the heart failure epidemic: prevalence, incidence rate, lifetime risk and prognosis of heart failure. The Rotterdam Study. Eur Heart J.

[16] Adams KF, Fonarow GC, Emerman CL (2005). Characteristics and outcomes of patients hospitalized for heart failure in the United States: rationale, design, and preliminary observations from the first 100,000 cases in the Acute Decompensated Heart Failure National Registry (ADHERE). Am Heart J.

[17] McMurray JJ, Adamopoulos S, Anker SD (2012). ESC guidelines for the diagnosis and treatment of acute and chronic heart failure 2012: the task force for the Diagnosis and Treatment of Acute and Chronic Heart Failure 2012 of the European Society of Cardiology. Developed in collaboration with the Heart Failure Association (HFA) of the ESC. Eur J Heart Fail.

[18] Yancy CW, Jessup M, Bozkurt B (2013). 2013 ACCF/AHA Guideline for the Management of Heart Failure: a report of the American College of Cardiology Foundation/American Heart Association Task Force on Practice Guidelines. J Am Coll Cardiol.

[19] Fonarow GC, Abraham WT, Albert NM (2008). OPTIMIZE-HF investigators and hospitals. Factors identified as precipitating hospital admissions for heart failure and clinical outcomes: findings from OPTIMIZE-HF. Arch Intern Med.

[20] Cheema B, Ambrosy AP, Kaplan RM (2018). Lessons learned in acute heart failure. Eur J Heart Fail.

[21] Nieminen MS, Brutsaert D, Dickstein K (2006). EuroHeart Failure Survey II (EHFS II): a survey on hospitalized acute heart failure patients: description of population. Eur Heart J.

[22] Roger VL, Go AS, Lloyd-Jones DM (2011). Heart disease and stroke statistics. 2011 update: a report from the American Heart Association. Circulation.

[23] Loehr LR, Rosamond WD, Chang PP (2008). Heart failure incidence and survival (from the Atherosclerosis Risk in Communities study). Am J Cardiol.

[24] Ray P, Birolleau S, Lefort Y (2006). Acute respiratory failure in the elderly: etiology, emergency diagnosis and prognosis. Crit Care.

[25] Kemp CD, Conte JV (2012). The pathophysiology of heart failure. Cardiovasc Pathol.

[26] English MA, Mastrean MB (1995). Congestive heart failure: public and private burden. Crit Care Nurs Q.

[27] Havranek EP, Abraham WT (1998). The health care economics of heart failure. Heart Fail.

[28] Viau DM, Sala-Mercado JA, Spranger MD (2015). The pathophysiology of hypertensive acute heart failure. Heart.

[29] Ural D, Çavuşoğlu Y, Eren M (2015). Diagnosis and management of acute heart failure. Anatol J Cardiol.

[30] Klabunde RE (2012). Chapter 9: Cardiovascular Integration, Adaptation, and Pathophysiology. Cardiovascular Physiology Concepts.

[31] Lloyd-Jones D, Adams RJ, Brown TM, Carnethon M, Dai S, de Simone G, Writing Group Members (2010). Heart disease and stroke statistics—2010 update: a report from the American Heart Association. Circulation.

[32] Heidenreich PA, Albert NM, Allen LA, Bluemke DA, Butler J, Fonarow GC (2013). Forecasting the impact of heart failure in the United States: a policy statement from the American Heart Association. Circ Heart Fail.

[33] Mohrman DE, Heller LJ, Weitz M, Boyle PJ (2010). Chapter 3: The Heart Pump. Cardiovascular Physiology.

[34] Lily LS, The faculty of the Harvard Medical School (2011). Chapter 9: Heart Failure. Pathophysiology of Heart Disease: A Collaborative Project of Medical Students and Faculty.

[35] Yancy CW, Jessup M, Writing Committee Members (2013). 2013 ACCF/AHA Guideline for the Management of Heart Failure: a report of the American College of Cardiology Foundation/American Heart Association Task Force on practice guidelines. Circulation.

[36] Anand IS, Florea VG (2001). High output cardiac failure. Curr Treat Options Cardiovasc Med.

[37] Mehta PA, Dubrey SW (2009). High output heart failure. QJM.

[38] Wasse H, Singapuri MS (2012). High-output heart failure: how to define it, when to treat it, and how to treat it. Semin Nephrol.

[39] Dolgin M, The Criteria Committee of the New York Heart Association (1994). Nomenclature and Criteria for Diagnosis of Diseases of the Heart and Great Vessels.

[40] Hunt SA, Baker DW, Chin MH (2001). ACC/AHA Guidelines for the Evaluation and Management of Chronic Heart Failure in the Adult: executive summary. A report of the American College of Cardiology/American Heart Association Task Force on Practice Guidelines (Committee to Revise the 1995 Guidelines for the Evaluation and Management of Heart Failure): developed in collaboration with the International Society for Heart and Lung Transplantation; Endorsed by the Heart Failure Society of America. Circulation.

[41] Ponikowski P, Voors AA, Anker SD (2016). 2016 ESC Guidelines for the diagnosis and treatment of acute and chronic heart failure: The Task Force for the diagnosis and treatment of acute and chronic heart failure of the European Society of Cardiology (ESC) Developed with the special contribution of the Heart Failure Association (HFA) of the ESC. EHJ.

[42] Chioncel O, Mebazza A, Harjola VP (2017). Clinical phenotypes and outcome of patients hospitalized for acute heart failure: the ESC Heart Failure Long-Term Registry. Eur J Heart Fail.

[43] Nohria A, Tsang SW, Fang JC (2003). Clinical assessment identifies hemodynamic profiles that predict outcomes in patients admitted with heart failure. J Am Coll Cardiol.

[44] Killip T, Kimball JT (1967). Treatment of myocardial infarction in a coronary care unit. A two year experience with 250 patients. Am J Cardiol.

[45] Forrester JS, Diamond GA, Swan HJ (1977). Correlative classification of clinical and hemodynamic function after acute myocardial infarction. Am J Cardiol.

[46] Collins S, Storrow AB, Kirk JD (2008). Beyond pulmonary edema: diagnostic, risk stratification, and treatment challenges of acute heart failure management in the emergency department. Ann Emerg Med.

[47] Gheorghiade M, Abraham WT, Albert NM (2006). Systolic blood pressure at admission, clinical characteristics, and outcomes in patients hospitalized with acute heart failure. JAMA.

[48] Braga JR, Tu JV, Austin PC (2013). Outcomes and care of patients with acute heart failure syndromes and cardiac troponin elevation. Circ Heart Fail.

[49] Flaherty JD, Bax JJ, De Luca L (2009). Acute heart failure syndromes in patients with coronary artery disease early assessment and treatment. J Am Coll Cardiol.

[50] Daniels LB, Laughlin GA, Clopton P (2008). Minimally elevated cardiac troponin T and elevated N-terminal pro-B-type natriuretic peptide predict mortality in older adults: results from the Rancho Bernardo Study. J Am Coll Cardiol.

[51] Wong YW, Fonarow GC, Mi X (2013). Early intravenous heart failure therapy and outcomes among older patients hospitalized for acute decompensated heart failure: findings from the Acute Decompensated Heart Failure Registry Emergency Module (ADHERE-EM). Am Heart J.

[52] Peacock WF, Emerman C, Costanzo MR (2009). Early vasoactive drugs improve heart failure outcomes. Congest Heart Fail.

[53] Collins SP, Lindsell CJ, Peacock WF (2006). The combined utility of an S3 heart sound and B-type natriuretic peptide levels in emergency department patients with dyspnea. J Cardiac Failure.

[54] Collins SP, Peacock WF, Lindsell CJ (2009). S3 detection as a diagnostic and prognostic aid in emergency department patients with acute dyspnea. Ann Emerg Med.

[55] Lokuge A, Lam L, Cameron P (2010). B-type natriuretic peptide testing and the accuracy of heart failure diagnosis in the emergency department. Circ Heart Failure.

[56] Robaei D, Koe L, Bais R (2011). Effect of NT-proBNP testing on diagnostic certainty in patients admitted to the emergency department with possible heart failure. Ann Clin Biochem.

[57] Wang CS, FitzGerald JM, Schulzer M (2005). Does this dyspneic patient in the emergency department have congestive heart failure?. JAMA.

[58] Wong GC, Ayas NT (2007). Clinical approaches to the diagnosis of acute heart failure. Curr Opin Cardiol.

[59] Martindale JL, Wakai A, Collins SP (2016). Diagnosing acute heart failure in the emergency department: a systematic review and meta-analysis. Acad Emerg Med.

[60] Abraham WT, Fonarow GC, Albert NM, OPTIMIZE-HF Investigators and Coordinators (2008). Predictors of in-hospital mortality in patients hospitalized for heart failure: insights from the Organized Program to Initiate Lifesaving Treatment in Hospitalized Patients with Heart Failure (OPTIMIZE-HF). J Am Coll Cardiol.

[61] Fonarow GC, Adams KF, Abraham WT, ADHERE Scientific Advisory Committee Study Group and Investigators (2005). Risk stratification for in-hospital mortality in acutely decompensated heart failure: classification and regression tree analysis. JAMA.

[62] Ambrosy AP, Vaduganathan M, Mentz RJ (2013). Clinical profile and prognostic value of low systolic blood pressure in patients hospitalized for heart failure with reduced ejection fraction: insights from the Efficacy of Vasopressin Antagonism in Heart Failure: Outcome Study with Tolvaptan (EVEREST) trial. Am Heart J.

[63] Opasich C, Rapezzi C, Lucci D (2001). Precipitating factors and decision-making processes of short-term worsening heart failure despite “optimal” treatment (from the INCHF Registry). Am J Cardiol.

[64] Ambardekar AV, Fonarow GC, Hernandez AF (2009). Get with the Guidelines Steering Committee and Hospitals. Characteristics and in-hospital outcomes for nonadherent patients with heart failure: findings from Get with the Guidelines-Heart Failure (GWTG-HF). Am Heart J.

[65] Renier W, Winckelmann KH, Verbakel JY (2018). Signs and symptoms in adult patients with acute dyspnea: a systematic review and meta-analysis. Eur J Emerg Med.

[66] Martindale JL (2016). Resolution of sonographic B-lines as a measure of pulmonary decongestion in acute heart failure. Am J Emerg Med.

[67] Mentz RJ, Kjeldsen K, Rossi GP (2014). Decongestion in acute heart failure. Eur J Heart Fail.

[68] Gheorghiade M, Follath F, Ponikowski P (2010). Assessing and grading congestion in acute heart failure: a scientific statement from the acute heart failure committee of the heart failure association of the European Society of Cardiology and endorsed by the European Society of Intensive Care Medicine. Eur J Heart Fail.

[69] Vyskocilova K, Spinarova L, Spinar J (2015). Prevalence and clinical significance of liver function abnormalities in patients with acute heart failure. Biomed Pap Med Fac Univ Palacky Olomouc Czech Repub.

[70] Aronson D, Burger AJ (2010). The relationship between transient and persistent worsening renal function and mortality in patients with acute decompensated heart failure. J Card Fail.

[71] Smith GL, Lichtman JH, Bracken MB (2006). Renal impairment and outcomes in heart failure: systematic review and meta-analysis. J Am Coll Cardiol.

[72] Hillege HL, Nitsch D, Pfeffer MA (2006). Renal function as a predictor of outcome in a broad spectrum of patients with heart failure. Circulation.

[73] Pascual-Figal DA, Casas T, Ordonez-Llanos J (2012). Highly sensitive troponin T for risk stratification of acutely destabilized heart failure. Am Heart J.

[74] Mayo DD, Colletti JE, Kuo DC (2006). Brain natriuretic peptide (BNP) testing in the emergency department. J Emerg Med.

[75] Hill SA, Booth RA, Santaguida PL (2014). Use of BNP and NT-proBNP for the diagnosis of heart failure in the emergency department: a systematic review of the evidence. Heart Fail Rev.

[76] Mueller C, Scholer A, Laule-Kilian K (2004). Use of B-type natriuretic peptide in the evaluation and management of acute dyspnea. N Engl J Med.

[77] Van Kimmenade RR, Pinto YM, Bayes-Genis A (2006). Usefulness of intermediate amino-terminal pro-brain natriuretic peptide concentrations for diagnosis and prognosis of acute heart failure. Am J Cardiol.

[78] Moe GW, Howlett J, Januzzi JL, Zowall H, Canadian Multicenter Improved Management of Patients With Congestive Heart Failure (IMPROVE-CHF) Study Investigators (2007). N-terminal pro-B-type natriuretic peptide testing improves the management of patients with suspected acute heart failure: primary results of the Canadian prospective randomized multicenter IMPROVE-CHF study. Circulation.

[79] Krauser DG, Lloyd-Jones DM, Chae CU (2005). Effect of body mass index on natriuretic peptide levels in patients with acute congestive heart failure: a ProBNP Investigation of Dyspnea in the Emergency Department (PRIDE) substudy. Am Heart J.

[80] McCullough PA, Duc P, Omland T (2003). B-type natriuretic peptide and renal function in the diagnosis of heart failure: an analysis from the Breathing Not Properly Multinational Study. Am J Kidney Dis.

[81] Anwaruddin S, Lloyd-Jones DM, Baggish A (2006). Renal function, congestive heart failure, and amino-terminal pro-brain natriuretic peptide measurement: results from the ProBNP Investigation of Dyspnea in the Emergency Department (PRIDE) Study. J Am Coll Cardiol.

[82] Levitt JE, Vinayak AG, Gehlbach BK (2008). Diagnostic utility of B-type natriuretic peptide in critically ill patients with pulmonary edema: a prospective cohort study. Crit Care.

[83] Bajaj A, Rathor P, Sehgal V (2015). Prognostic value of biomarkers in acute non-massive pulmonary embolism: a systematic review and meta-analysis. Lung.

[84] Coutance G, Cauderlier E, Ehtisham J (2011). The prognostic value of markers of right ventricular dysfunction in pulmonary embolism: a meta-analysis. Crit Care.

[85] Ruocco G, Cekorja B, Rottoli P (2015). Role of BNP and echo measurement for pulmonary hypertension recognition in patients with interstitial lung disease: an algorithm application model. Respir Med.

[86] Bergler-Klein J, Gyongyosi M, Maurer G (2014). The role of biomarkers in valvular heart disease: focus on natriuretic peptides. Can J Cardiol.

[87] Lam LL, Cameron PA, Schneider HG (2010). Meta-analysis: effect of B-type natriuretic peptide testing on clinical outcomes in patients with acute dyspnea in the emergency setting. Ann Intern Med.

[88] Trinquart L, Ray P, Riou B, Teixeira A (2011). Natriuretic peptide testing in EDs for managing acute dyspnea: a meta-analysis. Am J Emerg Med.

[89] Maisel A, Hollander JE, Guss D (2004). Primary results of the Rapid Emergency Department Heart Failure Outpatient Trial (REDHOT). A multicenter study of B-type natriuretic peptide levels, emergency department decision making, and outcomes in patients presenting with shortness of breath. J Am Coll Cardiol.

[90] Maisel AS, Krishnaswamy P, Nowak RM (2002). Rapid measurement of B-type natriuretic peptide in the emergency diagnosis of heart failure. N Engl J Med.

[91] McCullough PA, Nowak RM, McCord J (2002). B-type natriuretic peptide and clinical judgment in emergency diagnosis of heart failure: analysis from Breathing Not Properly (BNP) Multinational Study. Circulation.

[92] Roberts E, Ludman AJ, Dworzynski K (2015). The diagnostic accuracy of the natriuretic peptides in heart failure: systematic review and diagnostic meta-analysis in the acute care setting. BMJ (Clinical research ed).

[93] Moe GW, Howlett J, Januzzi JL, Zowall H (2007). N-terminal pro-B-type natriuretic peptide testing improves the management of patients with suspected acute heart failure: primary results of the Canadian prospective randomized multicenter IMPROVE-CHF study. Circulation.

[94] Rutten JH, Steyerberg EW, Boomsma F (2008). N-terminal pro-brain natriuretic peptide testing in the emergency department: beneficial effects on hospitalization, costs, and outcome. Am Heart J.

[95] Schneider HG, Lam L, Lokuge A (2009). B-type natriuretic peptide testing, clinical outcomes, and health services use in emergency department patients with dyspnea: a randomized trial. Ann Intern Med.

[96] Singer AJ, Birkhahn RH, Guss D (2009). Rapid Emergency Department Heart Failure Outpatients Trial (REDHOT II): a randomized controlled trial of the effect of serial B-type natriuretic peptide testing on patient management. Circ Heart Fail.

[97] Boldanova T, Noveanu M, Breidthardt T (2010). Impact of history of heart failure on diagnostic and prognostic value of BNP: results from the B-type Natriuretic Peptide for Acute Shortness of Breath Evaluation (BASEL) study. Int J Cardiol.

[98] Meisel SR, Januzzi JL, Medvedovski M (2012). Pre-admission NT-proBNP improves diagnostic yield and risk stratification – the NT-proBNP for EValuation of dyspnoeic patients in the Emergency Room and hospital (BNP4EVER) study. Eur Heart J Acute Cardiovasc Care.

[99] Steinhart BD, Levy P, Vandenberghe H (2017). A randomized control trial using a validated prediction model for diagnosing acute heart failure in undifferentiated dyspneic emergency department patients-results of the GASP4Ar study. J Card Fail.

[100] Dzudie A, Milo O, Edwards C (2014). Prognostic significance of ECG abnormalities for mortality risk in acute heart failure: insight from the Sub-Saharan Africa Survey of Heart Failure (THESUS-HF). J Card Fail.

[101] Knudsen CW, Omland T, Clopton P (2004). Diagnostic value of B-type natriuretic peptide and chest radiographic findings in patients with acute dyspnea. Am J Med.

[102] Václavík J, Špinar J, Vindiš D (2014). ECG in patients with acute heart failure can predict in-hospital and long-term mortality. Intern Emerg Med.

[103] Sartini S, Frizzi J, Borselli M (2017). Which method is best for an early accurate diagnosis of acute heart failure? Comparison between lung ultrasound, chest X-ray and NT pro-BNP performance: a prospective study. Intern Emerg Med.

[104] Kennedy S, Simon B, Alter JH (2011). Ability of physicians to diagnose congestive heart failure based on chest x-ray. J Emerg Med.

[105] Collins SP, Lindsell CJ, Storrow AB, Abraham WT, ADHERE Scientific Advisory Committee, Investigators and Study Group (2006). Prevalence of negative chest radiography results in the emergency department patient with decompensated heart failure. Ann Emerg Med.

[106] Volpicelli G, Melniker LA, Cardinale L (2013). Lung ultrasound in diagnosing and monitoring pulmonary interstitial fluid. Radiol Med.

[107] Deeb MA, Barbic S, Featherstone R (2014). Point-of-care ultrasonography for the diagnosis of acute cardiogenic pulmonary edema in patients presenting with acute dyspnea: a systematic review and meta-analysis. Acad Emerg Med.

[108] Price S, Platz E, Cullen L (2018). Echocardiography and lung ultrasonography for the assessment and management of acute heart failure. Nature Reviews Cardiology.

[109] Gargani L, Pang PS, Frassi F (2015). Persistent pulmonary congestion before discharge predicts rehospitalization in heart failure: a lung ultrasound study. Cardiovasc Ultrasound.

[110] Jambrik Z, Monti S, Coppola V (2004). Usefulness of ultrasound lung comets as a nonradiologic sign of extravascular lung water. Am J Cardiol.

[111] Ahmadpour H, Shah AA, Allen JW, Edmiston WA, Kim SJ, Haywood LJ (1983). Mitral E point septal separation: a reliable index of left ventricular performance in coronary artery disease. Am Heart J.

[112] Secko MA, Lazar JM, Salciccioli LA, Stone MB (2011). Can junior emergency physicians use E-point septal separation to accurately estimate left ventricular function in acutely dyspneic patients?. Acad Emerg Med.

[113] Silverstein JR, Laffely NH, Rifkin RD (2006). Quantitative estimation of left ventricular ejection fraction from mitral valve E-point to septal separation and comparison to magnetic resonance imaging. Am J Cardiol.

[114] Anderson KL, Jenq KY, Fields JM, Panebianco NL, Dean AJ (2013). Diagnosing heart failure among acutely dyspneic patients with cardiac, inferior vena cava, and lung ultrasonography. Am J Emerg Med.

[115] Gil Martinez P, Mesado Martinez D, Curbelo Garcia J, Cadinanos Loidi J (2016). Amino-terminal pro-B-type natriuretic peptide, inferior vena cava ultrasound, and bioelectrical impedance analysis for the diagnosis of acute decompensated CHF. Am J Emerg Med.

[116] Kajimoto K, Madeen K, Nakayama T, Tsudo H, Kuroda T, Abe T (2012). Rapid evaluation by lung-cardiac-inferior vena cava (LCI) integrated ultrasound for differentiating heart failure from pulmonary disease as the cause of acute dyspnea in the emergency setting. Cardiovasc Ultrasound.

[117] Miller JB, Sen A, Strote SR (2012). Inferior vena cava assessment in the bedside diagnosis of acute heart failure. Am J Emerg Med.

[118] Russell FM, Ehrman RR, Cosby K, Ansari A, Tseeng S, Christian E, Bailitz J (2015). Diagnosing acute heart failure in patients with undifferentiated dyspnea: a lung and cardiac ultrasound (LuCUS) protocol. Acad Emerg Med.

[119] Spinar J, Parenica J, Vitovec J (2011). Baseline characteristics and hospital mortality in the Acute Heart Failure Database (AHEAD) main registry. Crit Care.

[120] Long B, Koyfman A, Gottlieb M (2018). Management of heart failure in the emergency department setting: an evidence-based review of the literature. J Emerg Med.

[121] Stiell IG, Clement CM, Brison RJ (2013). A risk scoring system to identify emergency department patients with heart failure at high risk for serious adverse events. Acad Emerg Med.

[122] Stiell IG, Perry JJ, Clement CM (2017). Prospective and explicit clinical validation of the Ottawa Heart Failure Risk Scale, with and without use of quantitative NT-proBNP. Acad Emerg Med.

[123] Lee DS, Stitt A, Austin PC (2012). Prediction of heart failure mortality in emergent care: a cohort study. Ann Intern Med.

[124] Gil V, Miró Ò, Schull MJ (2018). Emergency Heart Failure Mortality Risk Grade score performance for 7-day mortality prediction in patients with heart failure attended at the emergency department: validation in a Spanish cohort. Eur J Emerg Med.

